# Abstracts of the 2017 A-CURE Symposium

**DOI:** 10.1007/s12265-017-9770-0

**Published:** 2018-01-16

## Overview and Mission of the 2017 A-CURE Symposium

The 2nd Annual Acute Cardiac Unloading and Recovery (A-CURE) Symposium was held in Barcelona, Spain, on August 25, 2017. Over 100 clinical and basic researchers from around the world participated in this year’s conference, which was hosted by the A-CURE Working Group. The mission of the A-CURE Working Group is to advance the science and mechanistic understanding of acute cardiac unloading and to support the translation of basic and clinical research into therapies aimed at heart muscle recovery. The last 20 years has seen major technical advances in mechanical circulatory support and research focused on pharmacological means to unload the heart. This progress has enabled new treatment modalities within the clinic for addressing myocardial infarction, cardiogenic shock, acute decompensated heart failure, and advanced heart failure. The goal of these approaches is to unload the heart of its metabolic demand and mechanical workload. Current research indicates that ventricular unloading has profound effects on both cardiac physiology and the cardioprotective processes associated with recovery after damage to the heart has taken place. Clinical and basic researchers from around the world have recognized the potential applications of this new technology to treat and understand several cardiac pathologies. The A-CURE Symposium is the only scientific conference dedicated to advancing the science and clinical application of acute unloading aimed at heart muscle recovery. The A-CURE initiative is expected to grow as the impact of this progressing science advances further into the clinic. This year’s keynote address by Dr. Valentin Fuster identified the far-reaching impact of myocardial infarction on the development of cardiovascular disease and heart failure. The lectures and abstracts presented during the 2nd annual meeting included basic and translational science and also presented cutting-edge clinical applications of acute unloading. The collection of selected abstracts presented here demonstrates the broad spectrum of research on display at the 2nd Annual A-CURE Symposium.

Navin Kapur, MD (Tufts Medical Center)

Bill O'Neill, MD (Henry Ford Hospital)

Dan Burkhoff, MD, PhD (Columbia University)

Mark Anderson, MD (Hackensack University Medical Center)

Co-Chairs, A-CURE Working Group

## Pre-Clinical Investigations

### Primary Left Ventricular Unloading with a Trans-Valvular Axial Flow Pump Limits Ischemic Heart Failure After Acute Myocardial Infarction

Lara Reyelt, Michele Esposito, Xiaoying Qiao, Kevin Morine, Shiva Annamalai, Courtney Boggins, Peter Natov, Hyunji Koo, Richard H Karas, Navin K Kapur

*Tufts Medical Center*

**Background:** Ischemic heart failure is a major cause of morbidity and mortality worldwide. We previously reported that compared to primary reperfusion (PR), first reducing myocardial oxygen demand by activating an acute mechanical circulatory support device while delaying coronary reperfusion primary unloading (PU) reduces myocardial damage in models of acute myocardial infarction (AMI).

**Hypothesis:** The purpose of this study was to explore the long-term outcomes associated with primary unloading before myocardial reperfusion compared to primary reperfusion alone.

**Methods:** Animals were randomized into two groups, the control arm (PR) and the intervention arm (PU). The PR cohort underwent 90 min of left anterior descending (LAD) occlusion followed by 120 min of reperfusion. The PU group underwent 90 min of LAD occlusion, followed by insertion and activation of an Impella CP pump, with an additional 30 min of LAD occlusion, followed by 120 min of reperfusion. Both groups were recovered and monitored for 28 days. After 28 days, animals underwent cardiac magnetic resonance imaging (CMR) with late gadolinium enhancement (LGE) to quantify final myocardial infarct size using a Philips Achieva 1.5 Tesla scanner and repeat cardiac catheterization. Animals were then euthanized, and myocardial infarct size was determined using TTC staining.

**Results:** Fourteen animals completed the ischemia-reperfusion phase of the protocol. Two animals in the PR group died within 6 h after reperfusion, and 12 animals survived to 28 days (PR, *n* = 6; PU, *n* = 6). Compared to PR, LV scar as determined by LGE (3.9 ± 3.2 vs 9 ± 3.7%, *p* = 0.03) and anatomic pathology (7.2 ± 4.9 vs 14.9 ± 4.1%, *p* = 0.02) was significantly lower after PU (Fig. 1A–B). CMR-derived LV volumes, LV ejection fraction, and LV mass were not different between groups. At 28 days, PU significantly increased cardiac output (4 ± 0.6 vs 2.6 ± 0.2 L/min, *p* = 0.0008) and LV stroke work (3302 ± 586 vs 2308 ± 291 mL mmHg, *p* = 0.009) more than PR. Compared with PR, levels of b-MHC, calcineurin, and BNP mRNA were lower; and SERCA mRNA levels were higher within the non-infarct zone after PU. At 28 days after AMI, circulating brain natriuretic peptide levels were lower after PU compared with PR (Fig. 1C).

**Conclusions:** PU reduces LV scar, improves LV function, and limits indices of maladaptive cardiac remodeling and circulating BNP levels 28 days after AMI. Studies exploring primary unloading as an approach to limit ischemic heart failure are required.

### LV Support Using Impella Relieves LA Stretch and Inhibits Atrial Arrhythmias Through Reduced Oxidative Stress

Kiyotake Ishikawa, Olympia Bikou, Shin Watanabe, Kenneth Fish, Lauren Leonardson, Roger Hajjar

*Mt. Sinai Hospital*

**Background:** The Impella, a left ventricle (LV)-to-aorta percutaneous ventricular assist device, supports systemic hemodynamics and unloads the LV. The impact of Impella support on the left atrium (LA) has not been clearly defined.

**Hypothesis:** LV support using Impella reduces LV end-diastolic pressure (EDP) and consequently reduces LA pressure. This leads to alleviation of LA stretch and inhibits atrial arrhythmias through attenuating stretch-induced oxidative stress.

**Methods:** Yorkshire pigs underwent myocardial infarction (MI) creation (*n* = 10) using a percutaneous approach. One to 2 weeks after MI, pigs underwent LV unloading using an Impella CP. A pressure-volume catheter was inserted into the LA using a trans-septal approach, and the LA pressure-volume loops were continuously monitored during the experiments. Atrial arrhythmia inducibility was examined by rapidly pacing the right atrium.

**Results:** After MI, pigs presented with reduced LV ejection fraction (EF), increased LVEDP, and increased LA volumes. LV unloading with Impella CP resulted in the reduction of the LVEDP, while mean LA pressure and maximum LA volume decreased proportionally to LVEDP. LA pressure-volume loops exhibited a pump-flow dependent left-downward shift. This was associated with a reduced LA active work and improved LA passive stiffness. Prior to Impella, 80% of the pigs were arrhythmia-inducible. However, only 30% of the pigs were inducible after the Impella LV support. Time to spontaneous termination of atrial arrhythmias was significantly shorter with Impella LV support, and this correlated with LA maximum volume as well as with LA active work. LA molecular analysis revealed that NOX2 expression was increased after MI, whereas Impella LV support reduced NOX2 levels together with the reduction in oxidative stress markers. These results suggest that Impella relieves LA stretch and reduces atrial arrhythmogenicity through inhibition of NOX2-dependent oxidative stress.

**Conclusions:** Impella reduces LA pressure, unloads LA, and reduces atrial arrhythmogenicity in a post-MI pig model.

### Mechano-Chronotropic Unloading in the Acute Phase of Myocardial Infarction Strikingly Reduces Infarct Size and Prevents Future Heart Failure

Genya Sunagawa, Keita Saku, Takahiro Arimura, Takuya Akashi, Takuya Kishi, Hiroyuki Tsutsui, Kenji Sunagawa

*Kyushu University*

**Background:** Acute myocardial infarction (MI) remains a leading cause of chronic heart failure (CHF). The presence of unsalvaged myocardium leads to cardiac remodeling and CHF. Reducing myocardial oxygen consumption (MVO2) in acute MI is known to reduce infarct size. We reported that the combination of LV mechanical unloading and chronotropic unloading (i.e., bradycardia) synergistically reduced MVO2 more than 60%. In this study, we examined if the combination therapy outperforms mechanical unloading alone in reducing MI size and preventing heart failure in the long-term. We used a transvascular LV assist device (Impella) for mechanical unloading and I_f_ channel inhibitor ivabradine (IVA) for chronotropic unloading.

**Methods:** In 18 anesthetized dogs, we ligated the coronary arteries for 180 min and then reperfused. We initiated Impella or Impella and IVA (1 mg/kg, IV) 60 min after the onset of ischemia and continued the support until 60 min after reperfusion. We allocated animals into three groups: no treatment (IR, *n* = 6), Impella (*n* = 6), and Impella + IVA (*n* = 6). Four weeks after MI, we compared LV function, heart failure parameters, and infarct size.

**Results:** All results were reported in the following order: IR, Impella, and Impella + IVA. In the acute phase of MI, heart rate in Impella + IVA was by far lower than other groups (142 ± 22, 144 ± 16, and 89 ± 13 bpm, *p* < 0.05, respectively). Four weeks after MI, Impella + IVA normalized LV end-systolic elastance (5.8 ± 2.0, 6.9 ± 1.5, and 13.0 ± 4.6 mmHg/ml, *p* < 0.05, respectively) and decreased LV end-diastolic pressure (7.7 ± 4.3, 4.4 ± 2.2, and 2.5 ± 2.4 mmHg, *p* < 0.05, respectively) and NT-proBNP (2858 ± 1412, 2084 ± 971, and 889 ± 499 pg/ml, *p* < 0.05, respectively). Impella + IVA strikingly reduced the infarct size more than 70% (≈ 1–4.3/15.6) (15.6 ± 4.2, 8.7 ± 3.9, and 4.3 ± 3.4%, *p* < 0.05, respectively).

**Conclusions:** Combination of LV mechanical unloading and a bradycardic agent synergistically reduced infarct size and prevented subsequent heart failure. This “mechano-chronotropic unloading” may serve as a powerful option to prevent CHF after MI.

### Paradoxical Mechano-Energetic Costs of Acute Mechanical Intra-Ventricular Unloading: LVAD Energetic Unloading, What We Know For Sure that Maybe Just Ain’t So

Carlos del Rio, S Bennett, J Noel-Morgan, SR Roof, EG Geist, BL Youngblood, E Ferris, Y Ueyama, PI McConnell.

*Myokardia, Natiowide Children’s Hospital*

**Background:** By design, intra-cardiac left ventricular assist devices (LVADs) support systemic hemodynamics and cardiac output (CO) while decreasing myocardial preload and stroke volume.

**Hypothesis:** Under certain conditions (e.g., partial unloading), LVAD-support could lead to paradoxical increases in the effective arterial elastance (Ea), hindering mechano-energetic unloading. This study tested the hypothesis by assessing the acute effects of LVAD-support on systemic hemodynamics, left-ventricular (LV) mechano-energetics, and oxygen consumption (MVO2) in vivo.

**Methods:** Healthy sheep (*n* = 12) were instrumented to assess MVO2 (coronary sinus/arterial sampling catheters and LCX coronary flow probe), systemic arterial pressures/CO, and LV pressure-volume relationships. Data before/after LVAD unloading with a continuous flow device (LVAD, *n* = 8) were compared to those obtained during partial vena cava occlusion (CTRL, *n* = 8); data were also collected when phenylephrine was administered to restore systemic hemodynamics (CTRL + PE). Heart rate was held constant by atrial pacing. Atrial natriuretic peptide (ANP) production was quantified.

**Results:** Partial LVAD-support (57 ± 4%) decreased LV preload (− 13 ± 2%), filling pressures (− 29 ± 7%), and SV (− 28 ± 5%) and preserved systemic/peak LV pressures (− 3 ± 2%) and CO (− 1 ± 1%). Both the estimated Ea (+ 40 ± 11%) and Ees (+ 33 ± 7%) increased with support. Despite marked reductions in SW (− 29 ± 5%) and PVA (− 31 ± 4%), both coronary flow (− 3 ± 4%) and MVO2 (+ 1 ± 2%) changed negligibly. Complete support (109 ± 9%) decreased LV pressures (− 33 ± 10%), normalizing Ea (− 1 ± 14%) but not Ees (+ 54 ± 12%) with moderate MVO2 reductions (− 13 ± 4%). Unsupported reductions in preload (CTRL) decreased MVO2 (− 39 ± 4%), Qlcx (− 53 ± 4%), and PVA (− 58 ± 4%), while Ea and Ees remained unchanged; pressure support (CTRL + PE) increased Ea and blunted the MVO2 reductions (− 7 ± 2%). LVAD-support altered the MVO2 vs PVA relationship and triggered ANP release.

**Conclusions:** These results suggest that intra-cardiac LVAD-support can trigger mechano-energetic alterations, paradoxically hindering the ability of an LVAD to energetically unload the ventricle. These observations could have important implications in the understanding of myocardial recovery under LVAD-support.

### Primary Left Ventricular Unloading Limits Infarct Size by Increasing Myocardial Levels of Stromal Derived Factor One Alpha (SDF1a) in Acute Myocardial Infarction.

Michele Esposito, Xiaoying Qiao, Yali Zhang, Shiva Annamalai, Lara Reyelt, Courtney Boggins, Kevin Morine, Richard H Karas, Navin Kapur

*Tufts Medical Center*

**Background:** Stromal derived factor 1 alpha (SDF1a) is a constitutively expressed cardioprotective chemokine that is rapidly degraded by proteases. We reported that compared to primary reperfusion (PR), first reducing myocardial oxygen demand by activating a trans-valvular pump (Impella CP) while delaying coronary reperfusion (primary unloading [PU]) reduces infarct size in acute myocardial infarction (AMI).

**Hypothesis:** We hypothesize that PU reduces proteolytic degradation of SDF1a thereby increasing SDF1a signaling via the receptor CXCR4 in AMI.

**Methods:** AMI was induced by occlusion of the left anterior descending artery (LAD) for 90 min in male swine (*n* = 4/group). In the PR group, the LAD was reperfused for 120 min. In the PU group, after 90 min of ischemia, an Impella CP was activated, and the LAD left occluded for an additional 30 min, followed by 120 min of reperfusion. Whole-transcript expression analysis was performed on RNA from the infarct zone using Porcine 1.0 ST microarrays and ConsensusPathDB programs. Quantitative polymerase chain reaction, western blots, and activity assays determined expression and activity of the SDF1a signaling pathway. Sham-operated LV samples served as controls.

**Results:** Compared to PR, PU reduced fibrotic and inflammatory gene expression including reduced transcript levels of matrix-metalloprotease-2 (MMP2), MMP9, and dipeptidyl peptidase-4 (DPP4). Compared to PR, PU increased SDF1a protein levels within the infarct zone. Gel zymography confirmed reduced activity levels of MMP2 and MMP9 within the infarct zone after PU, not PR. Compared to PR, PU attenuated DPP4 protein levels and activity and protein levels of CXCR7, an SDF1a sequestration receptor, within the infarct zone. To explore a functional role for SDF1a in PU, adult male swine received intra-coronary injections of AMD3100, a CXCR4 receptor antagonist, after Impella CP activation. Loss of CXCR4 activity attenuated cardioprotective signaling via Akt, Erk, and GSK3b and increased infarct size compared to vehicle-treated controls.

**Conclusions:** We introduce a novel mechanism by which PU limits proteolytic degradation and CXCR7 mediated sequestration of SDF1a, thereby increasing cardioprotective signaling via SDF1a and overcoming a critical barrier to SDF1a therapeutics in AMI.

### Neural Unloading by Intravascular Vagal Nerve Stimulation in Acute Myocardial Infarction Strikingly Reduces Infarct Size and Preserves Cardiac Function Despite Delaying Coronary Reperfusion

Keita Saku, Genya Sunagawa, Takahiro Arimura, Takuya Kishi, Hiroyuki Tsutsui, Kenji Sunagawa

*Center for Disruptive Cardiovascular Medicine, Kyushu University*

**Background:** Although early reperfusion is essential to save lives and prevent subsequent heart failure (HF) in AMI, the door-to-reperfusion time cannot be drastically shortened in the real-world setting. To overcome this limitation, we recently developed intravascular vagal nerve stimulation (iVNS) i.e., neural unloading and demonstrated that iVNS initiated 90 min after the onset of ischemia reduced infarct size by 61% and prevented HF in a dog model of ischemia/reperfusion. However, in the clinical setting, starting iVNS could be much later and introducing iVNS could delay reperfusion. Therefore, we examined the impact of iVNS under a more realistic clinical scenario. We started iVNS very late (180 min after the onset of ischemia), delayed reperfusion by 30 min, and examined its impact 4 weeks later.

**Methods:** In mongrel dogs, we occluded the LAD for 180 min to create ischemia and then reperfused for 60 min (control, *n* = 6). In iVNS (*n* = 7) 180 min after the LAD occlusion, we applied iVNS for 60 min and delayed the reperfusion by 30 min (210 min ischemia in total). We increased the intensity of iVNS to the maximum level where hemodynamics remained stable (7.5 ± 2.5 V, 10 Hz). Four weeks after AMI, we compared LV function and infarct size between the control group and dogs treated with iVNS.

**Results:** In comparison with control, despite a 30-min delay in reperfusion (longer ischemic period), iVNS increased LV ejection fraction (47.2 ± 5.4 vs 57.4 ± 4.1%, *p* < 0.01) and end-systolic elastance (3.8 ± 0.5 vs 10.0 ± 3.4 mmHg/ml, *p* < 0.01) and decreased LV end-diastolic pressure (17.9 ± 6.3 vs 4.5 ± 1.8 mmHg, *p* < 0.01). iVNS more than halved the infarct size (normalized by LV circumferential area, 13.3 ± 2.7 vs 6.1 ± 4.1%, *p* < 0.01).

**Conclusions:** iVNS initiated after the 180 min of ischemia prevented the worsening of LV function and markedly reduced infarct size despite delaying reperfusion. Therefore, neural unloading may serve as a therapeutic option in the management of AMI.

### Early Left Ventricular Unloading in Cardiogenic Shock Due to Acute Myocardial Infarction via Impella Microaxial Flow-Pump as an Impactful Device

Jan-Thorben Sieweke, Jörn Tongers, Christian Kühn, L. Christian Napp, Axel Haverich, Johann Bauersachs, Andreas Schäfer

*Hannover Medical School*

**Background:** Despite improvements in the management of cardiogenic shock (CS) complicating acute myocardial infarction (AMI), mortality remains unreasonably high. In the IABP-Shock II Trial, intra-aortic balloon counterpulsation did not affect mortality in CS patients. Larger prospective data on hemodynamic support strategies are still lacking.

**Methods:** We investigated 154 consecutive patients with CS between January 2013 and April 2016 who were supported by an Impella for isolated LV support at our department. In order to compare the efficacy of active LV unloading, we focused on patients fulfilling the inclusion/exclusion criteria of the IABP shock II Trial.

**Results:** Out of 154 consecutive patients, 125 were excluded (shock onset > 12 h, *n* = 40; ROSC > 30 min, *n* = 15; withdrawal life support, *n* = 6; biventricular unloading, *n* = 49; no intrinsic heart action, *n* = 10; cerebral deficit, *n* = 5). Compared to the IABP-Shock II Trial, our cohort (total *n* = 29; Impella 2.5, *n* = 4; Impella CP, *n* = 25 patients, mean age of 63.9 ± 2.2 years) exhibited similar baseline characteristics and a similar severity of CS as assessed by SAPS-II score (50.4 ± 1.6). In our registry, coronary disease state was severe with increased prevalence of prior PCI (44.8%), prior coronary artery bypass grafting (CABG, 17.2%) and a greater amount of patients with out-of-hospital cardiac arrest (55.2%) with return of spontaneous circulation after 14.2 ± 2.5 min. The safety outcome was reasonable as in-hospital re-infarction or thrombosis did not occur in our cohort. In the IABP Shock II Trial, 30-day mortality was 41.3% in the control group and 39.7% in the IABP group. In our patients, however, early usage of Impella was associated with a mortality rate as low as 20.7%.

**Conclusions:** These findings suggest that the Impella platform is a promising device for mechanical hemodynamic support in CS particularly due to AMI. To be able to interpret these findings more accurately, a propensity score matched analysis is needed. Therefore, we collaborated with the UKE Hamburg to perform a propensity score matched analysis in a larger collection of patients. Our registry data highlight the serious challenge of prospective studies to include enough patients to provide the study with sufficient statistical power. The Dan Shock trial is a promising prospective trial which is now extended into Germany (DanGer Shock trial).

### Left Ventricular Performance and Tissue Perfusion During Support with Veno-Arterial ECMO vs Impella CP in a Large Porcine Model of Profound Cardiogenic Shock

Ole Møller-Helgestad, Janus Adler Hyldebrandt*, Ann Banke, Charlotte Svejstrup Rud, Nanna Louise Junker Udesen, Louise Linde, Lisette Okkels Jensen, Henrik Schmidt, Hanne Berg Ravn#, Jacob Eifer Møller

*Odense University*

**Background:** The use of mechanical circulatory support, including Impella CP and veno-arterial extra corporeal membrane oxygenation (ECMO), is increasing in the treatment of cardiogenic shock (CS) following STEMI. However, studies comparing the technologies are lacking.

**Hypothesis:** A combination of norepinephrine and Impella CP is associated with unloading of the left ventricle (LV) compared to ECMO in a large porcine model of CS.

**Methods:** CS was induced by staged microembolization of the left main coronary artery in 12 female swine. A conductance catheter was placed in the LV for measurement of LV end-diastolic pressure (LVEDP), LV end-diastolic volume (LVEDV), and pressure-volume area (PVA). Arterial lactate levels were obtained at CS and 60 min of mechanical support. Data are presented as medians at CS and as relative percentages of CS at 60 min of support, with 95% confidence intervals. A two-sample Wilcoxon rank-sum test was used to identify differences between Impella CP and ECMO.

**Results:** In Impella CP (*n* = 6) vs ECMO (*n* = 6), cardiac output was 2.6 L/min (1.9; 3.2) vs 2.0 L/min (1.8; 3.3) *p* = 0.2, MAP 31 mmHg (24; 37) vs 35 mmHg (27; 42) *p* = 0.5, lactate 3.9 mmol/L (2.7; 5.3) vs 2.8 mmol/L (1.7; 5.0) *p* = 0.3, LVEDP 20 mmHg (12; 23) vs 16 mmHg (13; 26), *p* = 0.3, LVEDV 178 mL (137; 211) vs 160 mL (138; 172) *p* = 0.1, and PVA 2827 mmHg*mL (2023; 3460) vs 2594 mmHg*mL (1927; 2930) *p* = 0.3. At 60 min of mechanical support with Impella CP vs ECMO, relative changes from CS of LVEDP was 65% (47; 82) vs 113% [(92; 134) *p* = 0.007, LVEDV 63% (42; 83) vs 93% (78; 108) *p* = 0.03, PVA 113% (64; 162) vs 232% (179; 285) *p* = 0.02, MAP 184% (128; 240) vs 204% (160; 248) *p* = 0.5, and lactate 47% (31; 62) vs 78% [27; 129) *p* = 0.26.

**Conclusions:** A combination of Impella CP and norepinephrine ensures unloading of the LV compared with ECMO without compromising tissue perfusion in a large porcine model of CS

### Optimal Hemodynamic Support During Cardiac Arrest in the Cardiac Catheterization Laboratory

Kapildeo Lotun, Tam Truong, MD. Karl B Kern, MD

*University of Arizona*

**Background:** Cardiac arrest (CA) can occur in the cath lab during high-risk percutaneous coronary intervention. While attempting to correct the precipitating cause of CA, several options are available to maintain vital organ perfusion. These include manual chest compressions, often interrupted during the rescue PCI, or a continuously operating percutaneous left ventricular assist device (PLVAD).

**Methods:** Thirty swine (50–60 kg) were studied. The left main-proximal left anterior descending artery was occluded with a coronary balloon. Ventricular fibrillation (VF) was induced, and circulatory support was provided with either manual chest compressions (frequently interrupted) or the Impella™ 2.5 L PLVAD without any concurrent chest compressions. After 12 min, the balloon was deflated to restore coronary flow and defibrillation attempted at 16 min. Primary outcome was 24-h favorable neurological function (CPC 1 or 2), while secondary outcomes included defibrillation success, return of spontaneous circulation (ROSC), and hemodynamics.

**Results:** Manual chest compressions consistently produced higher systolic aortic and right atrial pressures, while Impella resulted in higher diastolic pressures. Coronary perfusion pressure (AoD-RaD) was not different between the manual and Impella-treated animals. Outcome measures consistently favored the Impella-treated group, though the small sample size limited the statistical differences.

**Table:** 

	Manual	Impella	*p*
Defib success	6/10	18/20	0.14
ROSC	5/10	16/20	0.12
24-hr CPC 1 or 2	0/10	7/20	0.06

**Conclusion:** Impella without chest compressions provides adequate hemodynamic support to resuscitate the majority of those suffering CA in the cath lab. Studies regarding the safety, hemodynamic efficacy, and outcome results with combining manual chest compressions and Impella will further elucidate the optimal approach for these patients.

### Inhaled Nitric Oxide Supports Myocardial Recovery Following Impella-Based Resuscitation

Andreas Ebeling, R. Zayat, A. Brücken, M. Fries, M. Derwall

*Hosptial RWTH Aachen*

**Background:** We have previously shown that survival after cardiac arrest can be improved by hemodynamically stabilizing a patient during CPR by using the minimally invasive left ventricular assist device, Impella 2.5 (iCPR). We have also demonstrated that the use of inhaled nitric oxide during iCPR leads to increased coronary perfusion, likely thru combined increased coronary pressure and iNO-dependent vasodilation. However, the role of iNO during Impella-supported myocardial recovery post-CPR is less clear.

**Hypothesis:** iNO fosters myocardial recovery by lowering pulmonary vascular resistance (PVRI).

**Methods:** Following electrically induced cardiac arrest and 9 min of untreated VF, eight pigs received iCPR and ventilation with an fiO_2_ of 1.0. At 1-h post return of spontaneous circulation (ROSC) and continuing for 5 h, animals inhaled oxygen at an fiO_2_ of 0.3 mixed with either 20 ppm iNO (*n* = 4, iNO) or nitrogen (*n* = 4, control). Hemodynamic parameters were determined using indwelling arterial and venous catheters.

**Results:** There were no significant differences regarding mean arterial pressure at baseline (BL), 10 min, 3 h, and 6-h post-ROSC iNO vs control, mmHg 98 ± 9 vs 92 ± 14; 76 ± 15 vs 90 ± 11; 76 ± 11 vs 83.3 ± 12.1; 76 ± 23 vs 75 ± 10). No differences were observed in Impella pump flow (10 min, 1.87 ± 0.2 L/min vs 2.2 ± 0.1; 1 h, 2.2 ± 0.1 vs 2.3 ± 0; 3 h, 2 ± 0.4 vs 2.3 ± 0.1; 5 h, 1.4 ± 0.2 vs 1.3 ± 0.3). Cardiac output was significantly higher during application of iNO, (BL, 7.1 ± 1.3 l/min vs 8.0 ± 0.4; 10 min, 8.4 ± 1.3 vs 8.7 ± 0.9; 3 h, 4.3 ± 0.8 vs 8.9 ± 2.5, *p* = 0.01; 6 h, 4.3 ± 0.9 vs 5.1 ± 2.2). This difference was not present after weaning from iNO. No significant difference in mean pulmonary arterial pressure (MPAP) among groups were observed at any time point. In contrast, pulmonary vascular resistance (PVR) was significantly reduced during the application of iNO (BL, 97 ± 14 dyn*sec*cm^− 5^ vs 65 ± 40; 10 min, 118 ± 48 vs 63 ± 28; 3 h, 381 ± 170 vs 44 ± 34, *p* = 0.004; 6 h, 278 ± 46 vs 274 ± 215).

**Conclusion:** Combining iCPR with iNO enhances the functional recovery of the heart in part by decreasing PVR without altering MPAP. Further echocardiographic studies are warranted to investigate the effects of iNO on myocardial contractility and unloading in this setting.

### Servo Controlled Sympathetic Unloading Markedly Augments Volume Tolerance and Instantly Resolves Pulmonary Congestion in Acute Heart Failure

Takeshi Tohyama, Keita Saku, Kazuya Hosokawa, Kamada Kazuhiro, Takuya Kishi, Hiroyuki Tsutsui, Kenji Sunagawa

*Kyushu University*

**Background:** Acute heart failure (AHF) excessively activates sympathetic nerve activity (SNA) which, in turn, increases stressed volume and left ventricular-end diastolic pressure (LVEDP), and often leads to hypertension and life-threatening pulmonary edema. Although sympathetic unloading is a reasonable therapeutic option to this pathophysiology, interventions to manipulate SNA without compromising hemodynamics remain unestablished. The purpose of this investigation was to develop a closed-loop neuromodulator which stimulates the carotid sinus (baroreceptor) nerves in response to changes in AP bionic baroreflex system (BBS). We examined if BBS suppresses SNA and rescues pulmonary congestion without inducing devastating hypotension in a rat model of AHF.

**Methods:** In six Sprague-Dawley rats with sino-aortic denervation, we created AHF by myocardial infarction. We attached electrodes to the right carotid bifurcation for neurostimulation and recorded AP and LVEDP. We servo controlled AP using BBS. In a volume tolerance study, we infused donated blood (0 to 10 ml/kg) stepwise. We compared AP and LVEDP between BBS and sham stimulation (SHAM). In the treatment study, we infused volume (10 ml/kg) and created acute pulmonary congestion. We activated the BBS and compared major hemodynamic responses with baseline.

**Results:** In the volume tolerance study, BBS reduced volume dependence of AP (11.0 ± 3.5 vs 5.5 ± 2.1 mmHg/ml/kg, *p* < 0.05) and LVEDP (3.72 ± 1.15 vs 2.48 ± 1.10 mmHg/ml/kg, *p* < 0.05) compared to SHAM, indicating marked improvement of volume tolerance. In the treatment study, BBS instantly suppressed SNA (− 49.5 ± 2.1%) and lowered LVEDP (20.5 ± 5.1 vs 10.8 ± 4.8 mmHg, *p* < 0.05), while excessively high AP returned toward the normal level (165.3 ± 14.0 vs 119.7 ± 8.8 mmHg, *p* < 0.05). The physiological AP was achieved by the servo-mechanism of BBS. The settling time (80% of maximum response) of LVEDP was extremely brief (5.2 ± 1.2 s).

**Conclusions:** BBS markedly improves volume tolerance and instantly (within seconds) resolves pulmonary congestion in AHF model rats. BBS may serve as a novel therapeutic strategy via sympathetic unloading for AHF.

### PTEN Inhibition with VO-OHPIC Ameliorates Myocardial Ischemia-Reperfusion Injury Mediated by RISK Pathway Activation-Mediated Reduction of Apoptosis: An In Vitro and In Vivo Study

Carlos Santos-Gallego, Kiyotake Ishikawa, Belen Picatoste, Ida Unhammer, Shin Watanabe, Torsten P. Vahl, Javier Sanz, Jagat Narula, Roger J. Hajjar, Valentin Fuster, Juan J. Badimon.

*Mt. Sinai Hospital*

**Background:** Mechanical unloading of the LV during STEMI reduces MI size; however, additional cardioprotective strategies complementary to unloading could even further decrease the size of MI. RISK pathway is the main cardioprotective pathway against ischemia reperfusion (IR) injury, and it starts with the phosphorylation (activation) of Akt. Phosphatase and tensin homolog deleted on chromosome ten (PTEN) is a primary phosphatase that dephosphorylates (deactivates) Akt.

**Hypothesis:** PTEN inhibition using the drug VO-OHPIC ameliorates IR injury using both in vitro and porcine models.

**Methods:** Human AC16 cardiomyocytes underwent hypoxia-reoxygenation; PTEN inhibition was achieved genetically (siRNA) or pharmacologically (VO-OHPIC) in the presence or absence of inhibitors of the RISK and SAFE cardioprotective pathways. In vivo IR was induced in pigs by 1-h balloon occlusion of proximal LAD; VO-OHPIC was administered 15 min pre-reperfusion. Ten pigs were sacrificed at 1-day post-IR to study mechanisms of cardioprotection. Twelve additional pigs were studied with magnetic resonance and 3D-echocardiography at 1 week and 1-month post-IR to assess cardioprotection and LV remodeling.

**Results:** In vitro, both genetic and pharmacological PTEN inhibition activated RISK (but not SAFE) pathway, increased cell viability, and reduced apoptosis (flow cytometry for annexin-V and caspase-3 activation). The benefits of PTEN inhibition were mediated via RISK activation because they were abrogated with RISK (but not SAFE) inhibitors. In short-term porcine experiments, PTEN inhibition activated RISK pathway, decreased cardiomyocyte apoptosis (TUNEL 17.1 ± 14.6 vs 44.3 ± 8.4, *p* = 0.02) and oxidative stress, improved mitochondrial function and myocardial energetics, and reduced infarct size (TTC 29.7 ± 2.6 vs 35.5 ± 2.7, *p* = 0.02). In long-term porcine IR experiments evaluated with cardiac MRI and 3D–echo, PTEN inhibition increased myocardial salvage (29.8 ± 4.4 vs 7.1 ± 3.8, *p* < 0.01), reduced infarct size (LGE 28.3 ± 1.7 vs 36.7 ± 2.6, *p* = 0.01), improved both systolic and diastolic function (EF 39.8 ± 4.1 vs 34.5 ± 3.7, *p* = 0.02; − dP/dt − 917 ± 4.1 vs − 505 ± 49, *p* = 0.01), and reduced adverse post-infarction cardiac remodeling (LVESV 57.8 ± 19 vs 73.4 ± 11, *p* < 0.05).

**Conclusions:** PTEN inhibition ameliorates IR injury both using in vitro and in vivo (porcine) models via enhancing the RISK pathway activity.

### Impact of Impella Support on Microvascular Perfusion of Cardiac and Major Organs

Shin Watanabe, Olympia Bikou, Kenneth Fish, Lauren Leonardson, Roger J. Hajjar, Kiyotake Ishikawa

*Mt. Sinai Hospital*

**Background:** Impact of Impella on systemic tissue perfusion in a post-MI setting remains poorly characterized.

**Hypothesis:** Impella support will increase tissue perfusion of major organs by increasing the systemic cardiac output.

**Methods:** Myocardial infarction was induced in Yorkshire pigs by occluding the left anterior descending (LAD) artery with a coronary balloon for 60 min followed by thrombus injection using a percutaneous method. Impella support was initiated for 120 min in two pigs immediately after acute myocardial infarction and four pigs 2 weeks after myocardial infarction. Changes in tissue perfusion of the infarct, infarct-border, and remote myocardium, as well as major organ perfusion were assessed by injecting fluorecent microspheres before and 120 min after Impella support.

**Results:** Impella support increased the cardiac output from 3.0 ± 0.5 to 3.5 ± 1.3 L/min. After Impella support for 120 min, infarct perfusion was significantly increased relative to the baseline perfusion (190 ± 89% relative to baseline, *p* = 0.01). In contrast, perfusion of the infarct border and remote did not show significant changes albeit the numerical increase (infarct border, 121 ± 44%, *p* = 0.22, remote 134 ± 74%, *p* = 0.27). Perfusion of the liver, lung, brain, spleen, and kidney also increased numerically; however, significant difference was found only in the brain perfusion (168 ± 36%, *p* = 0.05). Tissue perfusion of the infarct border, remote, liver, and kidney showed tight correlations, but that of the brain, lung, and spleen did not show significant correlation to any of cardiac and other major organs evaluated.

**Conclusion:** Impella increases infarct and brain perfusion in a pig model of MI. Cardiac non-infarct tissue, liver, and kidney showed similar patterns in the perfusion changes, whereas cardiac-infarcted tissue, brain, lung, and spleen showed independent patterns.

### Heterotopic Heart Transplantation in a Mouse Model to Study Mechanical Unloading—Establishment of MEA Measurements to Study Ventricular Arrhythmias

Sumi Westhofen, Leonie Dreher, Hermann Reichenspurner, Heimo Ehmke, Alexander Peter Schwoerer.

*UHZ Hamburg-Eppendorf*

**Background:** Unloading of failing hearts by assist devices can lead to negative alterations in cardiomyocyte physiology and lead to issues such as ventricular arrhythmias, which is associated with increased mortality and morbidity. Observations in patients and translational animal models indicate that reduction in workload itself induces impaired calcium handling and cellular electrophysiology. Pathophysiological relevance of these alterations is unclear.

**Hypothesis:** Heterotopic heart transplantation (hHTX) in transgenic mice allows detailed studies of pathophysiological alterations of unloading. Since the procedure is technically highly demanding with low error tolerance, it is hardly used. Multielectrode arrays (MEAs) are used to measure extracellular field potentials, up to mainly in brain slices. The establishment in hearts has been shown in rats and cell cultures but hardly in mice. We established MEA investigation of heterotopically transplanted mouse hearts to study electrophysiology and pathophysiology of unloaded cardiac tissue to allow a better understanding of the origin and propagation of the arrhythmic potentials.

**Methods:** The transplantation technique is based on internationally accepted hHTX models in rodents, leading to complete mechanical unloading. Slices of orthotopic and unloaded mouse hearts (300 μm) were cut with a microtome in a Tyrode’s solution containing 0.9 mM Ca2 + and BDM (2,3-Butanedione Monoxime), a contraction blocker. They were then transferred into a warm buffer solution without BDM and began to beat spontaneously. A MEA with 64 electrodes was used to measure electrical activity. Conduction velocity, propagation direction, and the QT interval were analyzed.

**Results:** Compared to orthotopic hearts of recipient animals, 14 days of unloading induced weight reduction of the graft by ~ 25%. Slices of orthotopic and unloaded hearts showed intact vitality up to 2 h. In both loaded and unloaded hearts, conduction velocity and QT intervals (range 65 ms–105 ms) could be measured with stable, reproducible results and showed a rate independency in a defined frequency span.

**Conclusions:** We present our first results establishing MEA measurements in unloaded mouse hearts. Our MEA data are reproducible and comparable to published data of action potential durations of adult mice hearts. MEA analysis of heterotopically transplanted mouse hearts is feasible, allowing studies of electrohysiological alterations in unloaded hearts.

### LV Unloading Using an Impella CP Improves Coronary Flow and Infarct Zone Perfusion in Ischemic Heart Failure

Olympia Bikou, Shin Watanabe, MD, Kenneth Fish, Lauren Leonardson, Roger J. Hajjar, Kiyotake Ishikawa

*Mt. Sinai Hospital*

**Background:** Increase in coronary flow after an ischemic insult may lead to an efficient delivery of therapeutic materials, such as genes and stem cells.

**Hypothesis:** LV unloading with the Impella CP increases coronary flow and myocardial perfusion (MP) in a sub-acute myocardial infarction (MI).

**Methods:** An anterior transmural MI (infarct size 26.1 ± 4.1%) was induced by occluding the proximal left anterior descending artery for 90 min in Yorkshire pigs (*n* = 9), followed by a thrombus injection. Two weeks after the MI, six animals underwent LV unloading with an Impella CP for 2 h, whereas three animals underwent vasodilator therapy using sodium nitroprusside (0.24 μg/Kg/h). Epicardial coronary flow by coronary flow wire, ventricular wall stress by pressure catheter and echocardiography, and MP using fluorescent microspheres were assessed.

**Results:** LV support with Impella CP increased total cardiac output (3.0 to 3.5 L/min). Impella significantly reduced end-diastolic volume (106 ± 17 to 89 ± 16 mL, *p* = 0.02) and end-diastolic pressure (27.6 ± 5.6 to 18.3 ± 6.6 mmHg), resulting in a significant decrease in LV end-diastolic wall stress (infarct zone 71.6 ± 14.7 to 43.3 ± 10.8 kdynes/cm^2^, *p* = 0.02; remote zone 66.6 ± 20.9 to 40.6 ± 13.3 kdynes/cm^2^, *p* = 0.02). Epicardial coronary flow increased acutely and remained elevated after 2 h. Compared to the baseline, MP measured by fluorescent microspheres significantly increased within the infarct zone (+ 109 ± 81%, *p* = 0.003), which correlated well with the end-diastolic wall stress (*r*^2^ = 0.54, *p* = 0.006). MP of the remote non-ischemic myocardium did not change significantly (+ 26 ± 74%, *p* = 0.84). Vasodilator treatment also reduced end-diastolic wall stress; however, epicardial coronary flow only increased transiently. Coronary flow did not increase in one pig that developed severe hypotension (< 60 mmHg).

**Conclusions:** LV unloading using an Impella CP decreased wall stress and increased coronary flow. MP was increased only in the infarct zone and correlated with the change in wall stress. This approach provides new opportunities to mechanically intervene coronary flow and myocardial perfusion after MI.

### Predictors of Impella-Induced RV Failure in Post-MI Pigs

Shin Watanabe, Olympia Bikou, Kenneth Fish, Lauren Leonardson, Roger J. Hajjar, Kiyotake Ishikawa

*Mt. Sinai Hospital*

**Background:** Right ventricle (RV) failure becomes apparent after left ventricular assist device (LVAD) implantation in some of the patients with advanced heart failure (HF).

**Hypothesis:** Temporal left ventricular (LV) support with an Impella after myocardial infarction (MI) can also cause RV failure in a selected patient population.

**Methods:** Myocardial infarction was induced in Yorkshire pigs (20–40 Kg) by percutaneously occluding the left anterior descending (LAD) (*n* = 18) or the LCx (*n* = 2). Animals were evaluated for LV and RV function 1–2 weeks after MI using echocardiography and a pressure catheter. Pigs were then implanted with the Impella CP, and the pump flow was increased stepwise.

**Results:** In ten pigs, LV collapsed while RV dilated significantly before reaching the maximum pump support, indicating an appearance of RV failure. Comparison of echocardiographic and hemodynamic parameters before Impella support in pigs with and without RV failure exhibited pulmonary artery acceleration time (102 ± 25 vs 128 ± 27 ms, *p* = 0.04 and RV failure pigs vs no-RV failure pigs, respectively), tricuspid annular plane systolic excursion (15.6 ± 3.6 vs 18.8 ± 2.4 mm, *p* = 0.04), LVEF (34.4 ± 10.5 vs 44.0 ± 7.0%, *p* = 0.03), mean right arterial pressure (5.0 ± 2.9 vs 2.2 ± 1.5 mmHg, *p* = 0.001), mean RV pressure (6.4 ± 2.0 vs 2.9 ± 2.0 mmHg, *p* = 0.001), LV maximum pressure (93 ± 18 vs 113 ± 21 mmHg, *p* = 0.04), LV dP/dt maximum (1204 ± 254 vs 1691 ± 490 mmHg/s, *p* = 0.01), LV dP/dt minimum (− 1243 ± 274 vs − 1739 ± 450 mmHg/s, *p* = 0.008), and LV stroke work (2.49 ± 1.36 vs 3.99 ± 1.20 mmHg*L, *p* = 0.02) as significantly different parameters between the groups. Two LCx infarct pigs did not develop RV failure. Multiple logistic regression analysis revealed mean RV pressure as an independent predictor of RV failure.

**Conclusion:** RV failure was more frequently observed in pigs with worse LV function and those with signs of RV functional impairment after temporal Impella support.

### A Mouse Model of Cardiac Recovery

Ana Cruz-Solbes, Keith Youker, Guillermo Torre-Amione, Arvind Bhimaraj

*Houston Methodist*

**Background:** Although the concept of recovery in humans through mechanical circulatory support (MCS) devices has been increasingly recognized, there is no established mouse model to study cardiac recovery from heart failure (HF). We have previously created a non-ischemic mouse model of HF using C57 male mice, NaCl, and L-NAME in drinking water, and surgically implanted angiotensin II infusion pumps in a 5-week protocol.

**Hypothesis:** Recovery after the onset of HF is possible in a non-ischemic mouse model.

**Methods:** We utilized our non-ischemic mouse model and maintained a 3-month recovery phase by removing the angiotensin II pump and returning drinking water to normal. Age-matched control mice were simultaneously followed. Heart weight, fibrosis, and echocardiographic parameters were measured and analyzed in a serial manner.

**Results:** Mice in the HF group had increased fibrosis and heart weight compared to matched controls (*p* < 0.05), while mice in the recovery group had fibrosis and heart weight comparable to age-matched controls (*p* = 0.6) with a reduction of the HF phenotype compared to the HF mice (*p* < 0.05). Also, left ventricular ejection fraction was significantly reduced to below 40% in the HF mice compared to control (*p* < 0.05), while this parameter seemed to improve back to normal in the recovery group (recovery vs control *p* = 0.5; recovery vs HF *p* < 0.05). Fractional shortening was also reduced in the HF mice compared to control (*p* < 0.05) and returned to normal levels in the recovery mice.

**Conclusions:** We have developed a mouse model of heart failure recovery by documenting the expression and resolution of the HF phenotype using the described protocol. This model will facilitate the study of mechanisms involved in recovery of HF in order to device interventions beyond mechanical unloading for a successful sustained cardiac recovery.

## Clinical Investigations

### Ventricular Unloading by Impella Support Reduces Pulmonary Congestion in Patients with Cardiogenic Shock

Julian Wiora, Angelika Uwarow, Sandra Büter, Christian Jung, Malte Kelm, Ralf Westenfeld, Patrick Horn

*Universitaetsklinikum Duesseldorf*

**Background:** In patients with cardiogenic shock, pneumonia due to pulmonary congestion and subsequent septic shock is associated with high mortality.

**Hypothesis:** Left ventricular (LV) unloading by a percutaneous left ventricular assist device (pVAD), Impella, in patients with cardiogenic shock reduces pulmonary congestion and the downstream rate of pneumonia.

**Methods:** In this retrospective study, we assessed pulmonary congestion in patients with cardiogenic shock who received the Impella pVAD (*n* = 30) compared to those who received intra-aortic balloon pump (IABP) support (*n* = 31). As primary endpoint, pulmonary congestion was assessed by calculating characteristic findings on the chest X-ray using the Halperin score. Scores ranged from 0 to a maximum of 390 points and were assessed in both groups before (pre-) and longitudinally [24 h (day 1), 72 h (day 3)] during support. The rate of pneumonia was assessed as a secondary endpoint.

**Results:** The groups (Impella vs IABP) did not differ in terms of age, SOFA score, Apache score, or basal serum lactate levels. Total fluid balance was calculated as positive during support and did not differ in both groups: + 2.4 ± 0.6 l vs + 1.8 ± 0.4 l at day 1 (*p* = 0.57), + 1.3 ± 0.7 l vs + 0.6 ± 0.4 l at day 2 (*p* = 0.86) and + 0.2 ± 0.7 l vs +0.3 ± 0.9 l at day 3 (*p* = 0.80). Pulmonary congestion as indicated with the Halperin score decreased post-implantation in patients with Impella support (pre 224 ± 14, day 1 178 ± 11, day 3 169 ± 12, *p* = < 0.01), but it not decrease in patients with IABP support (pre 212 ± 14, day 1 214 ± 10, day 3 206 ± 9, *p* = 0.42). The incidence of pneumonia did not differ between Impella group (57%, 17/30) and IABP group (74%, 23/31) (*p* = 0.18).

**Conclusions:** Left ventricular unloading by Impella support reduces pulmonary congestion in patients with cardiogenic shock despite performed positive fluid balance. Therefore, Impella support may facilitate treatment of congestive pneumonia in patients with cardiogenic shock, although prospective trials in larger patient cohorts are required.

### Myocardial Mitochondrial ROS Production Is Reduced in the Left Ventricle of Mechanically Unloaded Hearts

Daniel Scheiber, Elric Zweck, Tomas Jelenik, Patrick Horn, Udo Boeken, Diyar Saeed, Malte Kelm, Michael Roden, Julia Szendrödi, Ralf Westenfeld

*Heinrich-Heine University, Düsseldorf, Germany*

**Background:** Increased ventricular filling pressure and volume overload are hallmarks of advanced heart failure (HF). Previous studies have linked pressure and volume overload with alterations in myocardial substrate utilization and mitochondrial energy production in HF. It is well established that mitochondrial production of reactive oxygen species is increased in HF leading to oxidative stress. Mechanical ventricular unloading has been shown to induce protective reverse remodeling in myocardial tissues; however, little is known about the impact of ventricular unloading on myocardial mitochondrial energy metabolism. In this study, we compare mitochondrial reactive oxygen species (ROS) production and respiration in human hearts that were mechanically supported prior to explantation for heart transplantation (HTX) to unsupported hearts.

**Hypothesis:** We hypothesize that (1) chronic left ventricular unloading reduces myocardial mitochondrial ROS production, and (2) improves myocardial mitochondrial oxidative capacity in patients suffering from advanced HF.

**Methods:** We prospectively evaluated 11 patients undergoing HTX between October 2016 and May 2017 in this single-center trial. Myocardial tissue specimens were harvested from macroscopically scar-free areas of the left ventricle during heart explantation. Mitochondrial hydrogen peroxide emission was analyzed fluoroscopically in an Oxygraph-2k (OROBOROS INSTRUMENTS, Austria). Mitochondrial oxidative capacity on citrate cycle-derived substrates and fatty acids was analyzed by high-resolution respirometry.

**Results:** Eight patients had left ventricular assist device (LVAD) implantation prior to heart transplantation (LVAD-HTX, age 58.9 years; BMI 27.12.7; six ischemic heart disease and two dilated heart disease (DCM)), and three patients did not receive mechanical circulatory support prior to transplantation (HTX, 49.11 years; BMI 26.57.1; 3*DCM). Myocardial mitochondrial reactive oxygen species (ROS) production was significantly decreased in the LVAD-HTX group compared to the HTX group (1.40 1 vs 0.90 1 pmol/(s*mg); *p* < 0.0001). There were no differences in either maximal oxidative capacity on fatty acids or citrate cycle-derived substrates, or on uncoupled respiration or mitochondrial coupling efficiency between the groups.

**Conclusions:** These data suggest that ventricular unloading decreases myocardial mitochondrial ROS production in the setting of HF, whereas mitochondrial respiration remains unaffected. This mechanism may reveal a new target of ventricular unloading-dependent cardioprotection.

### The Impella Micro-Axial Flow Catheter Is Safe and Effective for the Treatment of Myocarditis Complicated by Cardiogenic Shock: an Analysis from the Global cVAD Registry

Shiva Annamali, Michele Esposito, Sudeep Kuchibhotla, Shelley Hall, Theodore Schreiber, William O’Neill, Navin K. Kapur.

*Tufts Medical Center*

**Background:** Myocarditis complicated by cardiogenic shock (Myo-CS) remains a complex clinical problem. The use of acute mechanical circulatory support devices for cardiogenic shock is growing.

**Hypothesis:** We explored the clinical utility of Impella trans-valvular micro-axial flow catheters in the setting of Myo-CS.

**Methods:** We retrospectively analyzed data from 21 sites within the cVAD registry, an ongoing multicenter voluntary registry at sites in North America and Europe that have used Impella in patients with myocarditis. Myocarditis was defined by endomyocardial biopsy in 34% (*n* = 11) or by clinical history without angiographic evidence of coronary disease (*n* = 23).

**Results:** A total of 34 patients who received Impella support for Myo-CS were identified. Mean age was 42 ± 17 years with 17 males, and a mean left ventricular ejection fraction (LVEF) on admission was 18 ± 10%. Baseline mean cardiac index was 1.8 ± 0.5 L/min/m^2^; pulmonary capillary wedge pressure was 25 ± 7 mmHg, and right atrial pressure was 22 ± 3 mmHg. Mean lactate was 27 ± 31 mg/dL. Additionally, 32% (*n* = 11) of patients required intra-aortic balloon pump, and 85% (*n* = 29) were requiring vasopressor/inotrope therapy prior to Impella placement. The Impella 2.5, CP, and 5.0 were used in 41% (*n* = 14), 35% (*n* = 12), and 24% (*n* = 8), respectively. Impella support was used for a mean duration of 90.56 ± 74.26 h. Mean LVEF improved from admission to discharge among myocarditis patients unsupported with Impella (18 + 10 to 37 + 20%, *p* = 0.001). In-hospital mortality was 38% (*n* = 13), and 47% (*n* = 16) of patients were discharged alive with 94% recovering without need for further device therapy (Figure below).

**Conclusions:** This is the largest analysis of Impella-supported myocarditis cases to date. The use of the Impella appears to be safe and effective in the settings of myocarditis complicated by cardiogenic shock.

### Impella Is Associated with Shorter Time-to-Support and Reduced Incidence of Contrast-Induced Nephropathy Compared to VAECMO in High-Risk PCI

Julian Wiora, Patrick Horn, Christian Jung, Susanne Wolters, Tobias Zeus, Malte Kelm, Ralf Westenfeld

Universitaetsklinikum Duesseldorf

**Background:** Demographics are shifting to an elderly population with more comorbidities, potentially qualifying for mechanical circulatory support (MCS) during high-risk percutaneous coronary intervention (PCI) in order to maintain coronary perfusion pressure and hemodynamic stability. In this cohort, acute kidney injury (AKI) occurs frequently and is associated with increased short- and long-term mortality. Here, we compared clinical feasibility and incidence of AKI during high-risk PCI supported with peripheral VAECMO and Impella, respectively, in a single-center observational registry.

**Hypothesis:** Time-to-MCS implementation of Impella MCS is less time consuming and reduces the incidence of AKI compared to peripheral VAECMO during MCS-supported high-risk PCI.

**Methods:** Patients scheduled for elective MCS-supported high-risk PCI were included. All patients were turned down for surgery. Allocation to VAECMO (*n* = 10) or Impella (*n* = 11) was quasi random due to site-specific restrictions on the daily availability of the VAECMO platform. We analyzed PCI success, duration, peripheral complications (combination of inguinal bleeding, AV-fistula, and aneurysma spurium), and contrast-induced nephropathy (CIN) defined as increase in serum creatinin by ≥ 0.3 mg/dl within 48 h.

**Results:** Patient characteristics did not differ with respect to age (years) (VAECMO 72 ± 4 vs Impella 76 ± 3; *p* = 0.38), prevalence of severely reduced LV-function (70 vs 100%; *p* = 0.1), diabetes mellitus (40 vs 54%; *p* = 0.67), GFR (63 ± 6 ml/min vs 69 ± 6; *p* = 0.503), days of hospitalization (13.6 ± 11.5 vs 12.6 ± 6.2; *p* = 0.9), and days on intense care unit (7.9 ± 7.4 vs 3.9 ± 3.4; *p* = 0.25). All patients were successfully revascularized, and the MCS was weaned at the end of the procedure. Device implantation time was significantly shorter in the Impella group (70 ± 21 min vs 48 ± 9; *p* = 0.029). Likewise, procedure time tended to be shorter in the Impella cohort (170 ± 29 min vs 141 ± 47; *p* = 0.149). Peripheral vascular complications were equally distributed (3 vs 3; *p* = 1). Importantly, despite similar risks for CIN as indicated by similar Mehran score (%, 32 ± 6 vs 35 ± 5; *p* = 0.6) and a similar exposure to contrast medium (270 ± 113 vs 240 ± 85 mL; *p* = 0.9), Impella-supported PCI was associated with a reduced incidence of CIN (%, (Impella® 9 vs 60; *p* = 0.02).

**Conclusions:** MCS-assisted high-risk PCI with Impella support is associated with a lower incidence of contrast-induced nephropathy and a shorter time to MCS.

### An Inspection of Hypothermic Mechanical Support Preceding Coronary Reperfusion in Patients with a Profound Cardiogenic Shock Complicating Anterior ST-segment Elevation Myocardial Infarction

Makoto Suzuki, Kenichi Hagiya, Ryosuke Higuchi, Itaru Takamisawa, Tetsuya Tobaru, Tetsuya Sumiyoshi, Mitsuaki Isobe

*Sakakibara Heart Institute*

**Background:** We investigated whether pre-PCI procedural hypothermic mechanical support may provide clinical advantages in patients with profound cardiogenic shock complicating anterior STEMI.

**Methods:** Of the 483 consecutive patients treated with PCI for a first anterior STEMI, including 31 patients with aborted sudden cardiac arrest in our database, a total of 37 consecutive patients with an anterior STEMI complicated with profound cardiogenic shock, defined as the presence of hyper-lactic acidemia (serum levels of lactate > 4 mmol/L), treated with mechanical circulatory support were identified. In accordance with a presence or absence of pre-PCI procedural therapeutic hypothermia, a prevalence of impaired myocardial tissue-level reperfusion, defined by angiographic myocardial blush grades 0 or 1 and in-hospital mortality were evaluated as the clinical outcomes.

**Results:** Demographic characteristics revealed the mean age was 70 ± 14 years, 65% male, 41% hypertension, 49% diabetes mellitus, and serum levels of lactate of 7.8 ± 3.9 mmol/L. Median time from symptom onset to PCI was 80 min, and hospital admission to PCI was 52 min. All patients underwent PCI with mechanical circulatory support, and 13 patients were treated with pre-PCI procedural therapeutic hypothermia with a target core temperature of 34°C. Despite a similar achievement of post-PCI procedural TIMI flow grade III, less incidence of impaired myocardial tissue-level reperfusion was found in those with therapeutic hypothermia (38 vs 75%, *p* = 0.037). Overall in-hospital mortality was 70%, which were composed of 6 patients with therapeutic hypothermia and 20 of 24 without therapeutic hypothermia (46 vs 83%, *p* = 0.028). A logistic analysis demonstrated pre-PCI procedural therapeutic hypothermia as a potent contributor to in-hospital survival benefits (*p* = 0.006).

**Conclusions:** The present study may provide a great possibility for pre-PCI procedural hypothermic mechanical support to ameliorate in-hospital clinical features in patients with a profound cardiogenic shock complicating anterior STEMI.

### Interleukin-1 Blockade Improves Left Ventricular Contractility in Acute Heart Failure Without Increasing Myocardial Work

Leo Buckley, Salvatore Carbone, Cory Trankle, Justin Canada, Claudia Oddi Erdle, Jessica Regan, Michele Viscusi, Dinesh Kadariya, Hayley Billingsley, Ross Arena, Antonio Abbate, Benjamin Van Tassell

*Virginia Commonwealth University*

**Background:** Interleukin-1 is a pro-inflammatory cytokine and a key driver of systemic inflammation in acute heart failure (HF). In murine models, IL-1-driven inflammation acts as a direct cardiosuppressant and blunts the response to pharmacologic isoproterenol stress testing. In humans, plasma C-reactive protein level, a measureable readout of IL-1 activity, correlates with exercise capacity, and IL-1 blockade improves exercise capacity in both systolic and diastolic HF.

**Hypothesis:** We hypothesized that IL-1 blockade improves LV contractility as measured by LV end-systolic elastance in acute HF.

**Methods:** Acute HF patients were randomized to IL-1 blockade with IL-1 receptor antagonist (anakinra) or placebo for 14 days during hospitalization (*n* = 13) or within 2 weeks post-discharge (*n* = 40). LV end-systolic elastance was measured non-invasively as the slope of the LV end-systolic pressure-to-volume relationship (i.e., LV end-systolic pressure divided by LV end-systolic volume). bIRNe in LV end-systolic elastance between baseline and 14 days in the placebo and anakinra groups was analyzed with the Wilcoxon rank sum test for within-group change over time.

**Results:** There were no significant differences between anakinra and placebo patients with respect to age (*p* = 0.31), gender (*p* = 0.79), or high-sensitivity C-reactive protein (*p* = 0.94). Between baseline and 14 days, median % change in LV end-systolic elastance was 12% (IQR, − 7 to 36%) in the anakinra group and 0% in the placebo group (IQR, − 14 to 19%) (*p* = 0.08 for between-group difference). In the anakinra group, but not the placebo group, LV end-systolic elastance increased significantly (anakinra 0.13 (IQR, − 0.07 to 0.27), *p* = 0.03 vs baseline; for placebo 0 (IQR, − 0.17 to 0.14), *p* = 0.90 vs baseline). Pressure-volume area, a measure of myocardial work, did not change in either group (*p* > 0.19 for anakinra and placebo).

**Conclusions:** In this small pilot study, anakinra significantly increased LV contractility without increasing myocardial work. These results are consistent with the hypothesized mechanism of action for IL-1 blockade in acute heart failure.

### Ventricular Unloading by Impella as Bridge to Surgery in a Case of Complicated Myocardial Infarction

Efstratios Katsianos, Georg Wolff, Manuel Stern, Christian Jung, Malte Kelm, Ralf Westenfeld

*University Hospital Duesseldorf, Department of cardiology*

**Background:** Ventricular septal defect (VSD) is a rare but often fatal complication following myocardial infarction (MI). Patients with a ruptured septum usually present with the precipitous onset of hemodynamic compromise. Mortality of infarct-related VSD remains high; however, it varies significantly depending on the timing of surgical repair. Patients who underwent surgery within 7 days of presentation displayed 54% mortality, while delayed repair was associated with reduced mortality (18%). So far, the impact of left ventricular unloading using percutaneous mechanical circulatory support (MCS) on post-MI VSD has not been investigated.

**Hypothesis:** We hypothesized that Impella support may facilitate delayed surgical repair in a patient with cardiogenic shock in acute MI complicated by VSD.

**Results:** Case report: A 57-year-old male presented to the emergency department with angina pectoris for the last 5 days. He appeared diaphoretic in imminent shock (lactate 3.5 mmol/L). The 12-lead ECG revealed ST-elevations (> 2 mm) in the frontal leads (V2–V5). After unsuccessful volume challenge, norepinephrine was started (0.05 μg/kg/min) and increased rapidly during his transfer to the catheterization laboratory (0.2 μg/kg/min). Coronary angiography revealed a complete, subacute occlusion of the proximal left anterior descending (LAD) and corresponding severely reduced left ventricular function with apical and septal akinesia. In light of the further hemodynamic deterioration (lactate 6.5 mmol/L), we performed an oximetry run revealing a VSD (initial Qp:Qs ratio 1:4). The decision was made to postpone surgical repair due to excessive mortality risk. Mechanical circulatory support with Impella CP was inserted, and the patient stabilized over the next days (lactate < 1.5 mmol/L). Nevertheless, intracardiac shunting increased (Qp:Qs ratio 2:3) over time, and a VSD repair with pericardial patch and left ventricular aneurysmectomy reconstruction was performed successfully on day 7. Postoperatively, the patient had an uneventful course and was discharged 15 days postoperatively.

**Conclusions:** This case provides evidence that mechanical circulatory support with Impella may support a strategy of deferred surgery in patients with cardiogenic shock complicated by VSD. Ventricular unloading may create a window for scar formation bridging to secure surgical repair.

### Percutaneous Left Atrial Unloading for Prevention of Pulmonary Edema and to Facilitate Ventricular Recovery Under Extracorporeal Membrane Oxygenation Therapy

Alexander Bernhardt, Mathias Hillebrand, MD; Yalin Yildirim, MD; Samer Hakmi, MD; Florian M. Wagner, MD; Stefan Blankenberg, MD; Hermann Reichenspurner, MD, PhD; Edith Lubos, MD

*University Heart Center Hamburg*

**Background:** Left-sided unloading during extracorporeal membrane oxygenation (ECMO) therapy is crucial for the prevention of pulmonary edema and the facilitation of ventricular recovery. We present a case of a 55-year-old male patient under ECMO therapy with pre-existing LV thrombus formation in need of ventricular unloading.

**Methods:** We implanted a 21 French TandemHeart Protekt Solo Transseptal cannula into the left atrium (LA) using a trans-septal approach via femoral vein. The cannula was connected to the venous line of the ECMO circulation. A flow probe and a clamp to reduce flow, if necessary, were attached to the LA line. LA flow was adjusted to 900 ml/min under transesoephageal echocardiography (TEE) control to keep the atrial septum in the midline and prevent suction of the inflow cannula.

**Results:** After 9 days, the ECMO and TandemHeart cannula have to be weaned off stepwise and explanted. The patient was in NYHA class II without neurological sequelae (Cerebral Performance Scale 1). After 3 months, the patient has fully recovered and is working on a daily basis. The LV function is still moderately impaired and the LV thrombus is of the same size. The atrial septum showed no residual defect.

**Conclusions:** Percutaneous transseptal insertion of the TandemHeart cannula incorporated in an ECMO circulation for the prevention of pulmonary oedema and subsequent weaning from extracorporeal circulation was feasible and safe in a patient with cardiogenic shock and LV thrombus.

### Isolated Left Ventricular Failure Predicts Poor Outcome in Patients Receiving Veno-Arterial Extracorporeal Membrane Oxygenation

CA den Uil, LS Jewbali, AE Engstrom, AA Constantinescu, NM van Mieghem

*Erasmus Medical Center*

**Background:** Veno-arterial extracorporeal membrane oxygenation (VAECMO) is increasingly and successfully used to assist patients with refractory cardiogenic shock from different causes; however, it is currently not known which patients would benefit most from ECMO support.

**Hypothesis:** Since ECMO may hamper left ventricular unloading and worsen left ventricular pressure/volume relations, the presence of predominant left ventricular failure in patients on VAECMO support may be associated with worse outcome. To study this hypothesis, we investigated outcome according to the presence of isolated left vs isolated right vs biventricular heart failure in patients with refractory cardiogenic shock of different causes.

**Methods:** This single-center study included 132 patients with acute myocardial infarction (20%), acute on chronic heart failure (14%), post-cardiotomy (17%), cardiac allograft failure (8%), pulmonary embolism (16%), and acute non-ischemic heart failure (25%). Ventricular function was a priori assessed by transthoracic echocardiography (isolated left (26%), isolated right (22%), or biventricular heart failure (52%)). The primary endpoint was all-cause mortality at 90 days and long-term. Predictors for adverse outcome were identified by univariate and multivariate Cox regression analysis.

**Results:** Median duration of ECMO support was 6 (3–9) days. Ninety-day survival was 51% (isolated LV failure 32% vs isolated RV failure 62% vs biventricular failure 55%, *p* = 0.04). The main reason for withdrawal of ECMO support in patients with isolated LV failure was formation of extensive LV thrombus. The presence of isolated left ventricular failure was a predictor for 90-day mortality, irrespective of diagnosis and SAVE score. In patients who survived 90 days following ECMO implantation, long-term (4-year) survival was excellent (95%, no difference between subgroups).

**Conclusions:** Isolated left ventricular failure was an independent predictor for dismal 90-day outcome. This finding questions the routine use of VAECMO in patients with refractory pure LV failure. Pure LV ventricular unloading (by means of last-generation dedicated Impella pumps) might be more suitable because these devices reduce afterload, decrease LV volumes, and thus actively unload the ventricle.

### Early Biventricular Unloading Enables Safe Stabilization and Rescue of Patients with Refractory Cardiogenic Shock: Identification of Predictors of Success

Jörn Tongers, Jan-Thorben Sieweke, Christian Kühn, L. Christian Napp, Axel Haverich, Johann Bauersachs, Andreas Schäfer

*Hannover Medical School*

**Background:** Little progress has been made lately in cardiogenic shock (CS), and its mortality remains substantial in refractory CS. Hence, the evolving era of mechanical support is promising, although many aspects regarding the management of mechanical unloading remain uncertain.

**Hypothesis:** We evaluate efficacy and prognostic predictors of early biventricular unloading in refractory CS.

**Results:** In our single-center registry, 69 consecutive patients were analyzed (age 57 ± 2 yrs, 78% male). All patients required dual unloading by means of VAECMO plus Impella CP due to refractory CS (cardiomyopathy 45%, STEMI 41%, NSTEMI 15%, arrhythmias 1%). At admission and during the ICU course, patients were severely sick (SAPS-II 53 ± 13, SOFA 12 ± 3, 33% out-of-hospital CPR, 94% ventilation, 56% dialysis, 38% resuscitation). Impella microaxial pump and VAECMO were inserted and removed percutaneously via femoral access (duration Impella and vaECMO, range 55–182 vs 81–259 h). Reflecting clinical stabilization in response to mechanical support, number and dosing of catecholamines was rapidly reduced, while systolic arterial pressure increased (for all end/time points, *p* < 0.05. As a measure of the microcirculation, lactate levels normalized within 24 h (for all time points, *p* < 0.05 vs BL). After censoring patients in whom ICU therapy was withdrawn because of their living will, 30-day mortality was considerably low (40%). Univariate Cox regression revealed shock duration until first device, infection, RV-failure, SAPS-II and SOFA scores, and lactate level after 12 h as variables determining success of unloading therapy. Shock duration until first device (cut-off 13.5 h), infection, and lactate levels after 12 h (cut-off 3.25) predicted outcome in multivariate analysis. In addition, biventricular failure significantly predisposed negative outcome.

**Conclusions:** In conclusion, early biventricular unloading employing the Impella microaxial pump and VAECMO enables stabilization and rescue of refractory CS patients. Shock duration until initiation of support therapy, lactate levels at 12 h, and infections are multivariate predictors of CS survival.

### Unloading the Mystery: Lactate Clearance Is a Prognostic Marker for Survival in Cardiogenic Shock Receiving Early Impella Support

Shailendra Singh, Yonathon Litwok, Evelina Grayver, Perwaiz Meraj, Alexander Lee, Cindy Grines

*Northwell Health System*

**Background:** The survival of patients with acute myocardial infarction complicated by cardiogenic shock (AMICS) remains low despite early revascularization. The evolving percutaneous mechanical circulatory support (MCS) device options provide the possibility of a brighter future; however, markers are needed to assist in therapeutic decision-making when utilizing MCS in AMICS.

**Hypothesis:** We investigated if early dynamic changes in lactate levels would reflect the therapeutic benefit of mechanical support and its potential impact on survival in patients presenting with AMICS. We hypothesized that earlier MCS implantation would result in improved lactate clearance and overall survival.

**Methods:** Forty-six patients who presented to our coronary care unit underwent PCI and required MCS with an Impella 2.5 or Impella CP were included in this analysis. Differences between survivors and non-survivors were examined.

**Results:** Mean patient age was 63.7 ± 11.2, 85% were men, and mean left ventricular ejection fraction was 27 ± 11.7%. Less use of vasopressors was found in the survival group (1.8 vs 3.1; *p* < 0.0001). Adjunctive use of IABP had no impact on survival (*p* < 0.0866). Compared to pre-MCS lactate levels, survival demonstrated a significant decrease of 33.3% in lactate level obtained at 6 h, where as non-survivors had a slight increase in lactate level of 1.8% (*p* < 0.0001). Patients who had a lactate clearance of ≥ 30% at 6 h were 94% Survivors (*p* < 0.0001). Earlier MCS implantation was clearly exhibited in the survival group (1.0 vs 23.5 h, respectively; *p* < 0.0097). In fact, survival was 82.4% (*p* < 0.0037) when the Impella device was implanted within 2 h of admission to the hospital.

**Conclusions:** Patients who present with AMICS, serial lactate measurements may be used as a prognostic marker and therefore guide therapeutic decision-making. Early LV unloading with the Impella device and subsequent clearance of lactate improves survival in AMICS.

### Evolution of Biventricular Loading Condition in Pediatric LVAD Patient: a Prospective and Observational Study

Arianna Di Molfetta, R. Iacobelli, S. Filippelli, G. Grutter, G. Perri, F. Iodice, L. Pasquini, P. Guccione, A. Amodeo

*Hospital Bambino Gesu, Rome*

**Background:** Heart recovery in LVAD patients is related to reverse remodeling and unloading.

**Hypothesis:** The aim of this study was to describe the echocardiographic trend of LV and RV function after pulsatile flow LVAD in pediatrics.

**Methods:** From December 2013 to December 2016, we prospectively enrolled pediatric LVAD patients collecting clinical and echocardiographic data at the baseline, within 24 h after implantation and monthly until LVAD explant. Data were also used to calculate ventricular energetics parameters such as ventricular elastances, internal and external work, arterial elastance, and heart efficiency.

**Results:** Thirteen patients affected by DCM were enrolled (12.4 ± 11.0 months; 7.0 ± 3.6 kg). Average LVAD stay was 226.2 ± 121.2 days, 70% were successfully transplanted, and 30% died. LV end-systolic/end-diastolic volumes/end systolic-pressure volume relationship were significantly reduced until 2 months follow-up (*p* = 0.019/*p* = 0.001/*p* = 0.002), but then they start to worsen over time. RV function improved in the acute phase, but then it started to decrease over time with a progressive RV enlargement. Since the 4-month follow-up, RV fractional area change (RVFAC) worsening was related with the deterioration of LV unloading (*p* = 0.0036). LV atero-ventricular coupling significantly decreased after the LVAD (*p* = 0.04). LV/RV mechanical efficiency is improved/worsened (*p* = 0.02) by the LVAD.

**Conclusion:** Pulsatile LVAD in pediatrics is followed by an early and mid-term LV unloading that does not remain stable over time. RV function improved in the acute phase, but a progressive dilatation of RV was noted over time. The potential of heart recovery seems to be more promising during the early phase post LVAD.

### Percutaneous VAD in Children Supported with ECMO for Cardiogenic Shock

Sebastian Tume, Athar Qureshi, Dhaval Parekh, Antonio Cabrera, Ricardo Pignateli, Carlos Mery, Henri Justino, Iki Adachi

*Texas Children’s Hospital*

**Background:** Veno-arterial extracorporeal membrane oxygenation (VA-ECMO) increases afterload, which can result in pulmonary edema and/or ventricular distention if used in a heart with diminished systolic function. A catheter-based percutaneous ventricular assist device (PVAD) has recently emerged as an option for ventricular decompression during ECMO support. Conversely, it is not rare for patients with progressive cardiogenic shock on PVAD support to require additional support with ECMO. Experience with combined PVAD and ECMO support in children is limited. We present our initial experience of such an approach.

**Methods:** We conducted a retrospective chart review of all patients that underwent PVAD support in the setting of peripheral VA-ECMO at our institution. Data are presented as median (range).

**Results:** Six patients having age 13 (6.5–19) years, weight 51 (22–74) kg, and BSA 1.46 (0.91–1.97) m^2^ met our inclusion criteria: biventricular (*n* = 5) and univentricular physiology (*n* = 1). PVAD support was with Impella® 2.5 (*n* = 2) and CP (*n* = 4). While PVAD was placed using the femoral artery in all patients, ECMO was initiated via the central cannulation (*n* = 1), femoral (*n* = 4), or neck (*n* = 1) vessels. In four patients, ECMO was added 6 (0–15) hours after PVAD initiation to optimize perfusion. In the other two patients, PVAD was added after ECMO support for ventricular decompression, resulting in 6% reduction in LVEDD (35 to 33 mm) in one patient and 32% reduction in PCWP (19 to 13 mmHg) in the other. Duration of simultaneous PVAD and ECMO support was 6 (5–12) days. Hemolysis markers at 5 days of combined support remained in clinically acceptable ranges: LDH 1632 (559–10,274) U/L and plasma free hemoglobin 33 (30–150) mg/dL. There were, no major complications. However, PVAD insertion site bleeding was common (83%). In all patients, ECMO was weaned off after 6.5 (5–12) days of support, with PVAD remaining in place. Subsequently, PVAD support was discontinued 0.5 (0.5–5) days after ECMO decannulation. All patients survived support, and two patients expired at 7 and 23 days after support.

**Conclusions:** We describe the feasibility of PVAD as an adjunctive therapy to VA-ECMO support in children. The risk profiles of the combined support are not deemed substantially higher than those for ECMO alone. Its potential advantages include ventricular decompression and facilitation of ECMO weaning process. Further studies are warranted to clarify the safety and efficacy of such an approach in the pediatric population.

### Use of Trans-Carotid IMPELLA 2.5 Axial Flow Pump Device for Left Ventricle Unloading During VA-ECMO Support in Pediatric Acute Heart Failure

Gianluigi Perri, A. Di Molfetta, S. Filippelli, M.G.Gagliardi, A. Rizza, P. Guccione, A. Amodeo

*Hospital Bambino Gesu, Rome*

**Background:** Veno-arterial extracorporeal membrane oxygenation (VA-ECMO) is an established mechanical circulatory support (MCS) used to treat acute heart failure (HF) in adults and pediatric patients; however, the long-term efficacy is limited by the lack of direct left ventricular (LV) unloading.

**Hypothesis:** We present a trans-carotid Impella 2.5 implantation technique in a pediatric patient with peripheral VA-ECMO.

**Methods:** A 15-year-old boy (40 Kg, BSA 1.2 m^2^) was admitted for acute rejection after HF with a progressive cardiogenic shock requiring peripheral VA-ECMO. The patient developed progressive central venous pressure (CVP) increase with inadequate LV unloading, severe pulmonary edema, and progressive renal failure. It had been decided to implant a microaxial Impella 2.5 left ventricular assist device (LVAD) through a surgical trans-carotid approach because of its limited femoral artery size.

**Results:** A right cervicotomy was performed, and the medial and lateral border of sternocleidomastoid muscle were dissected, and carotid sheet was incised to expose the right common carotid artery (CCA) that was approximately 6 mm. A PTFE tube graft was end-to-side anastomosed to the CCA. The 2.5 Impella was inserted through the graft in the right CCA and advanced across the aortic valve inside the LV under fluoroscopic and transesophageal echocardiographic guidance. The ECMO support was initially maintained at 2.8 L/min and Impella at 1.4 L/min. Then, it progressively increased to 3 L/min and 1.9 L/min, respectively. Ten days later, the patient was successfully transplanted.

**Conclusion:** This is the first report of transcarotid percutaneous Impella implantation in a pediatric patient. Placement of a Goratex graft is an easy technique to optimize the insertion of the Impella 2.5 axial flow pump via CCA and minimize or avoid the vessel complications and inadequate cerebral flow. This approach might be an alternative and feasible option in pediatrics patients, affected by severe LV failure, as bridge to decision, or bridge to candidacy.

### Secondary LV Unloading After Transportation in Patients After Out-of-Hospital ECLS Implantation Improved Survival and Probability of Successful Weaning

Hermann Reichenspurner, Alexander Bernhardt

*University Heart Center Hamburg*

Secondary Left Ventricular (LV) Unloading After Transportation in Patients After Out-of-Hospital Extracorporeal Life Support (ECLS) Implantation Improved Survival and Probability of Successful Weaning

**Background:** ECLS is an established and recommended treatment modality in patients with acute carcinogenic shock. Safety and feasibility of this technique increased this treatment to out-of-hospital implantation and transportation. LV unloading in patients on ECLS has shown to increase survival and probability of successful weaning from short-term mechanical circulatory support (MCS). Here, we present our treatment algorithms and outcomes after ECLS implantation and transportation outside our own center.

**Methods:** Between October 2014 and March 2017, a total number of 47 consecutive patients (mean age 53 years, range 37 to 74) received ECLS in a referring institution. Of those, 29 (61.7%) received arterial-venous (VA) ECLS implantation using a femoral percutaneous approach. Prospective collected data were retrospectively analyzed for survival and weaning depending on LV unloading.

**Results:** The 30-day survival in the VA ECLS patients was 41.4%. Nine patients received an additional Impella 2.5 device for LV unloading after a mean time of 7.6 h, and one patient received an LA cannula after ECLS implantation and transportation to our center. Survival in patients with LV unloading was higher compared to those without LV unloading (60.0 vs 31.6%, *p* = 0.02). Approximately 50% of LV-unloaded patients were weaned from MCS vs 31.6% without additional LV unloading (*p* = 0.08). Two patients on ECLS and Impella 2.5 support were successfully bridged to Impella 5.0 support, with one patient weaned off later and one patient bridge to a durable assist device.

**Conclusions:** Secondary Impella 2.5 implantation in patients who have successfully received ECLS in a referring hospital improved survival and weaning from the short-term device and is therefore recommended in transported ECLS patients.

### Contemporary Trends in Cardiogenic Shock: Incidence, Intra-aortic Balloon Pump Utilization, and Outcomes

Krishnaraj S Rathod, Sudheer Koganti, M. Bilal Iqbal, Ajay K Jain, Zoe Astroulakis, Roby Rakhit, Miles C. Dalby, Tim Lockie, Iqbal S. Malik, Charles J Knight, Mark Whitbread, Anthony Mathur, Simon Redwood, Philip A. MacCarthy, Constantinos O’Mahony, Andrew Wragg, Dan Jones

*St. Bartholomew’s Hospital*

**Background:** Cardiogenic shock (CS) remains a major cause of morbidity and mortality in patients with ST-segment elevation myocardial infarction (STEMI). We aimed to assess the current trends in CS management, looking specifically at the incidence, use of intra-aortic balloon pump (IABP) therapy, and outcomes in patients with STEMI treated with primary percutaneous coronary intervention (PCI).

**Methods:** We undertook an observational cohort study of 21,210 STEMI patients treated between 2005 and 2015. Patients’ details were recorded at the time of the procedure into local databases using the British Cardiac Intervention Society (BCIS) PCI dataset. In this dataset, there were 1890 patients who presented with cardiogenic shock. Primary outcome was all-cause mortality at a median follow-up of 4.1 years (IQR range 2.2–5.8 years).

**Results:** Increasing rates of cardiogenic shock were seen over the course of the study with consistently high mortality rates of 45–70%. Exactly 685 patients underwent IABP insertion during primary PCI for CS with decreasing rates over time. Those patients undergoing IABP therapy were younger, more likely to have poor LV function, and less likely to have had previous PCI compared to the control group. Procedural success rates were similar (86.0 vs 87.1%, *p* = 0.292) although crude, in-hospital major adverse cardiac events (MACE) rates were higher (43.8 vs 33.7%, *p* < 0.0001) in patients undergoing IABP therapy. Kaplan-Meier analysis demonstrated significantly higher mortality rates in patients receiving IABP therapy (50.9% IABP vs 39.9% control, *p* < 0.0001) during the follow-up period. After multivariate Cox analysis (HR 1.01 95% CI 0.57–1.77) and the use of propensity matching (HR 1.35 95% CI 0.84–1.61), IABP therapy was not associated with mortality.

**Conclusions:** Cardiogenic shock treated by PCI is increasing in incidence and remains a condition associated with high mortality and limited treatment options. IABP therapy was not associated with a long-term survival benefit in this cohort and may be associated with increased early morbidity.

### Axillary Impella 5.0 As a Bridge to Durable LVAD

Arvind Bhimaraj

*Houston Methodist* *.*

**Background:** Patients in cardiogenic shock and multiorgan failure are considered too sick for a direct durable left ventricular assist device (LVAD) placement. Impella 5.0 can provide sufficient support in this setting and can be used as a bridge to durable LVAD placement.

**Hypothesis:** Impella 5.0 can be used as a bridge for durable LVAD.

**Methods:** We performed a single-center retrospective review of all consecutive patients supported with Impella 5.0 from August 2014 to October 2016 and identified those bridged to a durable LVAD.

**Results:** A total of 36 Impella 5.0 devices were implanted through an axillary graft in our institution. Of those, seven were used as a bridge for a durable LVAD (HMII). The main indication for implant was acute decompensated heart failure on chronic heart failure (5/7), and two were postsurgical (TAVR and CABG). The majority were Caucasian males with a median age of 61.2. Median length of hospital stay was 43 days, and median length of Impella support was 12 days (range 4–31). All patients had MCS at the time of Impella implant:six6 had IABP and one had VA ECMO. No pump dysfunction or replacement was seen for the duration of Impella support; only one patient required pump repositioning. All patients were ambulating and working with physical therapy at the time of durable LVAD implant. Four (4/7) survived to discharge post LVAD surgery.

**Conclusions:** Implantation of Impella 5.0 through axillary graft can be used as a strategy to stabilize patients in severe cardiogenic shock and improve their candidacy for a durable LVAD-support.

### Mobility Level During Acute Mechanical Circulatory Support with Axillary Deployment of the Impella 5.0 Is Associated with Improved Survival in Advanced Heart Failure and Shock

Shiva Annamalai, Michele Esposito, Xiaoying Qiao, Yali Zhang, Lara Reyelt, Courtney Boggins, Kevin Morine, Richard H Karas, Navin Kapur

*Tufts Medical Center*

**Background:** Mobility is an important indicator of recovery for patients with cardiogenic shock (CS). No studies have quantified peak mobility for patients with CS who are supported with the Impella 5.0 acute mechanical circulatory support (AMCS) device.

**Hypothesis:** The purpose of our study was to evaluate mobility levels among patients with CS being treated with an axillary Impella 5.0 pump.

**Methods:** We retrospectively analyzed data from 19 patients receiving an Impella 5.0 device for CS at our institution from 2013 to 2016. We used the Johns Hopkins Highest Level of Mobility (JH-HLM) Scale to quantify maximum mobility level achieved during active Impella 5.0 support. Higher scores on a scale of 1 to 8 indicated more mobility. Activity Measure for Post Acute Care (AM-PAC) Score was quantified for each patient to assess activity limitations, with a maximum score of 24.

**Results:** The mean age of the total cohort was 60 ± 12 years, and the mean left ventricular ejection fraction (LVEF) was 16 ± 6%. In-hospital mortality was 47% (*n* = 9). Of the 19 Impella 5.0 implants, 10 survived, 6 died from withdrawal of care, and 3 died from worsening heart failure/cardiogenic shock. Baseline clinical characteristics were similar between groups (Table 1). Of the survivor cohort, indications for device therapy were acute decompensated heart failure (ADHF; *n* = 7), ST-segment elevation myocardial infarction (STEMI; *n* = 1), and myocarditis (*n* = 2). Of the non-survivor cohort, indications included ADHF (*n* = 4), STEMI (n = 4), and post-cardiotomy CS (n = 1). Similar rates of mobilization during the time of Impella implant were seen between groups. Compared to non-survivors, survivors achieved a higher maximum JH-HLM level but similar AM-PAC scores (Figure).

Conclusion: Maximum mobility level after Impella 5.0 implantation may be associated with improved survival. The clinical utility of exercise as a therapeutic intervention for patients requiring prolonged AMCS requires further study. Based on these findings, we will report the design of the *Lazarus I* prospective study of functional status after Impella 5.0 implantation.

### Acute Biventricular Interaction in Pediatric Patients with Continuous or Pulsatile Flow LVAD: a Simulation Study.

Arianna Di Molfetta, Gianfranco Ferrari, Roberta Iacobelli, Sergio Filippelli, Gianluigi Perri, Antonio Amodeo.

*Hospital Bambino Gesu, Rome*

**Background:** Left ventricular assist devices (LVADs) are used to bridge pediatric patients to transplantation; however, the effects of LVADs on right ventricular (RV) function is controversial.

**Hypothesis:** This work aims at studying the ventricular interdependency in the presence of continuous (c-) and pulsatile (p-) flow LVAD in pediatric patients using a lumped parameter model including the representation of the septum.

**Methods:** Five pediatric patients’ data were used to simulate patients’ baseline. The effects on LV and RV functions, energetics, preloads, and afterloads of different c-LVAD speeds, p-LVAD rate, p-LVAD systole duration, p-LVAD filling, and ejection pressures were simulated.

**Results:** c-LVAD and p-LVAD unload the LV decreasing the LV external work and improving the LV ventriculo-arterial coupling, and these effects are more evident increasing the c-LVAD speed and the p-LVAD rate. c-LVAD and p-LVAD decrease the RV afterload, increase the RV ejection fraction, and improve the RV ventriculo-arterial coupling. The changes in RV function are inversely proportional to the degree of the interventricular septum leftward shift that increased by increasing the LVAD contribution.

**Conclusion:** The study of the interventricular interaction could lead to the development of a dedicated algorithm to optimize LVAD setting in pediatric population.

### Short- and Long-Term Changes in Ventricular Loading Conditions in LVAD Patients: Pulsatile vs Continuous Flow LVAD

Arianna Molfetta, L. Casadio, L. Fresiello, R. Iacobelli, S. Filippelli, G.Perri, G. Ferrari, P. Guccione, S. Jacobs, B. Meyns, M. Massetti, A. Amodeo

*Hospital Bambino Gesu, Rome*

**Background:** Heart remodeling during left ventricular assist device (LVAD) support is one important fact that can influence the possibility of heart recovery.

**Hypothesis:** We aimed at studying the evolution of left ventricular (LV) and right ventricular (RV) loading condition in LVAD patients comparing pulsatile (PVAD) and continuous flow (CVAD) LVAD.

**Methods:** Clinical and echocardiographic data of 19 PVAD pediatric patients were retrospectively collected and compared to literature data of 35 CVAD young adult patients to assess the changes in ventricular loading from baseline to follow up of 1, 3, and 6 months.

**Results:** Mean values of left ventricular ejection fraction improved from 22 ± 15 to 32.8 ± 13.9% in PVAD and from 21.1 ± 9.5 to 46 ± 20.6% in CVAD. In both cases, the major ejection fraction increment occurred during the first 30 days of LVAD. In CVAD, LV end-diastolic and end systolic diameters (LVEDD and LVESD, respectively) decreased progressively over time from 58.4 ± 22.8 to 52.7 ± 9.3 mm (9%, *p* = ns) and from 54.3 ± 21.2 to 40.1 ± 13.8 (26.1%, *p* = 0.04) in 6 months. In PVAD, LVEDD and LVESD decreased significantly at 1-month follow-up from 46.4 ± 9.2 to 34.2 ± 6.6 mm (26.2%, *p* = 0.000001) and from 40.9 ± 10.5 to 29.2 ± 7.4 mm (28%, *p* = 0.00001), but then a new LV enlargement was observed coming almost at the same diameters measured at baseline. In CVAD, RV function and dimensions decreased progressively, while RV dimensions progressively increased in PVAD, and RV function started to deteriorate since the 3-month follow-up.

**Conclusion:** At short-term follow-up, the LV unloading is higher in PVAD than in CVAD population. On the contrary, at long-term follow–up, the LV unloading provided by the CVAD is higher than in the PVAD population. Finally, RV function and dimensions are improved by CVAD, while they are worsened by PVAD.

